# Annual Report of the Bureau of Health of the Philippine Islands

**Published:** 1908-06

**Authors:** 


### ANNUAL REPORT OF THE BUREAU OF HEALTH OF THE PHILIPPINE ISLANDS.

The report of the Bureau of Health of the Philippines for the year ended June 30, 1907, by Victor G. Heiser, M. D. Director of Health, demonstrates that the sanitary reforms which have been persistently carried out during the past few years are bringing concrete and substantial results which more than justify their continuation. There has not been a single case of plague in the Islands and not one death from small pox in the city of Manila.

Since the method of combatting plague has been based upon the theory that its eradication could be accomplished by isolating the sick and destroying plague-infected rats, the efforts of the Bureau have met with complete success.

Four hundred and three deaths were reported as caused by [illegible]riberi. It is thought that this disease is more common among persons who eat only Philippine rice which is not husked until shortly before using, owing to the mold which soon renders it unfit for consumption, than among persons who use imported China or India rice, which has been husked for a year or more before being used.

A special effort is now being made to combat tuberculosis. During the past year in Manila one-sixth of the deaths were due to this cause. A bulletin has been issued on tuberculosis which discusses the symptoms and signs of the disease, and urges the importance of early diagnosis, especial stress being placed upon the importance of its prevention.

Typhoid fever is becoming more prevalent and seems to be obtaining a firmer foothold in the Philippines that it has had in the past.

Attention is called to the low per cent.of insanity in the Islands. It is one-fifth of that of Great Britain showing that it is far less prevalent than elsewhere. The report says that these figures are particularly significant in view of the fact that consanguineous marriages are common in the Philippines, since such marriages have been ascribed as one of the principle causes of mental derangement. It will be interesting to note in the future whether the increased nervous tension which naturally accompanies a higher civilization will be conducive to an increase in the proportion of insanity.

Owing to an increase in the number of deaths from amoebic dysentery, arrangements have been made to have instructions as to prophylaxis taught in the public schools.

By segregating the lepers at the Culion leper colony it is thought that the Islands may soon be freed from this disease. In collecting lepers from the Islands of Samar and Leyte for the purpose of transferring them to the Culion colony, it was found that errors had been made in the diagnosis and that many of the cases were tropical ulcers, phagadenas, or syphilis. The spirochaeta pallida was demonstrated in many of the patients.

Particularly satisfactory is the report on small pox. In the provinces where heretofore there have been more than 6,000 deaths annually from this one disease alone, not a single case of small pox has been reported since vaccination was completed more than a year ago.

